# Does developing multiple-choice Questions Improve Medical Students’ Learning? A Systematic Review

**DOI:** 10.1080/10872981.2021.2005505

**Published:** 2021-12-31

**Authors:** Youness Touissi, Ghita Hjiej, Abderrazak Hajjioui, Azeddine Ibrahimi, Maryam Fourtassi

**Affiliations:** a Faculty of Medicine and Pharmacy of Rabat, Mohammed V University, Souissi, Rabat, Morocco; b Faculty of Medicine and Pharmacy of Oujda, Mohammed Premier University, Oujda, Morocco; c Laboratory of Neurosciences, Faculty of Medicine and Pharmacy of Fes, Sidi Mohammed Ben Abdallah University, Fez, Morocco; d Laboratory of Biotechnology, Mohammed V University, Souissi, Rabat, Morocco; e Faculty of Medicine of Tangier, Abdelmalek Essaadi University, Tetouan, Morocco

**Keywords:** Multiple-choice-questions, learning, medical students, medical education

## Abstract

Practicing Multiple-choice questions is a popular learning method among medical students. While MCQs are commonly used in exams, creating them might provide another opportunity for students to boost their learning. Yet, the effectiveness of student-generated multiple-choice questions in medical education has been questioned. This study aims to verify the effects of student-generated MCQs on medical learning either in terms of students’ perceptions or their performance and behavior, as well as define the circumstances that would make this activity more useful to the students. Articles were identified by searching four databases MEDLINE, SCOPUS, Web of Science, and ERIC, as well as scanning references. The titles and abstracts were selected based on a pre-established eligibility criterion, and the methodological quality of articles included was assessed using the MERSQI scoring system. Eight hundred and eighty-four papers were identified. Eleven papers were retained after abstract and title screening, and 6 articles were recovered from cross-referencing, making it 17 articles in the end. The mean MERSQI score was 10.42. Most studies showed a positive impact of developing MCQs on medical students’ learning in terms of both perception and performance. Few articles in the literature examined the influence of student-generated MCQs on medical students learning. Amid some concerns about time and needed effort, writing multiple-choice questions as a learning method appears to be a useful process for improving medical students’ learning.

## Introduction

Active learning, where students are motivated to construct their understanding of things, and make connections between the information they grasp is proven to be more effective than passively absorb mere facts [1]. However, medical students, are still largely exposed to passive learning methods, such as lectures, with no active involvement in the learning process. In order to assimilate the vast amount of information they are supposed to learn, students adopt a variety of strategies, which are mostly oriented by the assessment methods used in examinations [2].

Multiple-choice questions (MCQs) represent the most common assessment tool in medical education worldwide [3]. Therefore, it is expected that students would favor practicing MCQs, either from old exams or commercial question banks, over other learning methods to get ready for their assessments [4]. Although this approach might seem practical for students as it strengthens their knowledge and gives them a prior exam experience, it might incite surface learning instead of constructing more elaborate learning skills, such as application and analysis [5].

Involving students in creating MCQs appears to be a potential learning strategy that combines students’ pragmatic approach and actual active learning. Developing good questions, in general, implies a deep understanding and a firm knowledge of the material that is evaluated [6]. Writing a good MCQ requires, in addition to a meticulously drafted stem, the ability to suggest erroneous but possible distractors [7,8]. It has been suggested that creating distractors may reveal misconceptions and mistakes and underlines when students have a defective understanding of the course material [6,9]. In other words, creating a well-constructed MCQ requires more cognitive abilities than answering one [10]. Several studies have shown that the process of producing questions is an efficient way to motivate students and enhance their performance, and linked MCQs generation to improve test performance [11–15]. Therefore, generating MCQs might develop desirable problem-solving skills and involve students in an activity that is immediately and clearly relevant to their final examinations.

In contrast, other studies indicated there was no considerable impact of this time-consuming MCQs development activity on students’ learning [10] or that question-generation might benefit only some categories of students [16].

Because of the conflicting conclusions about this approach in different studies, we conducted a systematic review to define and document evidence of the effect of writing MCQs activity on students learning, and understand how and under what circumstances it could benefit medical students, as to our knowledge, there is no prior systematic review addressing the effect of student-generated multiple-choice questions on medical students’ learning.

## Methods

### Study design

This systematic review was conducted following the guidelines of the Preferred Reporting Items for Systematic Review and Meta‐Analyses (PRISMA) [17]. Ethical approval was not required because this is a systematic review of previously published research, and does not include any individual participant information.

### Inclusion and exclusion criteria

Table 1 summarizes the publications’ inclusion and exclusion criteria. The target population was undergraduate and graduate medical students. The intervention was generating MCQs of all types. The learning outcomes of the intervention had to be reported using validated or non-validated instruments. We excluded studies involving students from other health-related domains, those in which the intervention was writing questions other than MCQs, and also completely descriptive studies without an evaluation section of the learning outcome. Comparison to other educational interventions was not regarded as an exclusive criterion because much educational research in the literature is case-based.

**Table 1.** Inclusion & exclusion criteria

	Inclusion criteria	Exclusion criteria
Population	Under-graduate/graduate medical students	Other health-related students: biomedical, nursing, and dental students
Intervention	Writing MCQs	Writing questions other than MCQs
Outcome	learning in terms of perceptions, performance, behavior	Entirely descriptive papers without an evaluation section

### Search strategy

On May 16^th,^ 2020, two reviewers separately conducted a systematic search on 4 databases, ‘Medline’ (via PubMed), ‘Scopus’, ‘Web of Science’ and ‘Eric’ using keywords as (Medical students, Multiple-choice questions, Learning, Creating) and their possible synonyms and abbreviations which were all combined by Boolean logic terms (AND, OR, NOT) with convenient search syntax for each database (Appendix 1). Then, all the references generated from the search were imported to a bibliographic tool (Zotero®) [18] used for the management of references. The reviewers also checked manually the references list of selected publications for more relevant papers. Sections as ‘Similar Articles’ below articles (e.g., PubMed) were also checked for possible additional articles. No restrictions regarding the publication date, language, or origin country were applied.

### Study selection

The selection process was directed by two reviewers independently. It started with the screening of all papers generated with the databases search, followed by removal of all duplicates. All papers whose titles had a potential relation to the research subject were kept for an abstract screening, while those with obviously irrelevant titles were eliminated. The reviewers then conducted an abstract screening; all selected studies were retrieved for a final full-text screening. Any disagreement among the reviewers concerning papers inclusion was settled through consensus or arbitrated by a third reviewer if necessary.

### Data collection

Two reviewers worked separately to create a provisional data extraction sheet, using a small sample made of 4 articles. Then, they met to finalize the coding sheet by adding, editing, and deleting sections, leading to a final template, implemented using Microsoft Excel® to ensure the consistency of collected data. Each reviewer then, extracted data independently using the created framework. Finally, the two reviewers compared their work to ensure the accuracy of the collected data. The items listed in the sheet were article authorship and year of publication, country, study design, participants, subject, intervention and co-interventions, MCQ type and quality, assessment instruments, and findings.

### Assessment of study methodological quality

There are few scales to assess the methodological rigor and trustworthiness of quantitative research in medical education, to mention the Best Medical Education Evaluation global scale [19], Newcastle–Ottawa Scale [20], and Medical Education Research Study Quality Instrument (MERSQI) [21]. We chose the latter to assess quantitative studies because it provides a detailed list of items with specified definition, solid validity evidence, and its scores are correlated with the citation rate in the succeeding 3 years of publication, and with the journal impact factor [22,23]. MERSQI evaluates study quality based on 10 items: study design, number of institutions studied, response rate, data type, internal structure, content validity, relationship to other variables, appropriateness of data analysis, the complexity of analysis, and the learning outcome. The 10 items are organized into six domains, each with a maximum score of 3 and a minimum score of 1, not reported items are not scored, resulting in a maximum MERSQI score of 18 [21].

Each article was assessed independently by two reviewers; any disagreement between the reviewers about MERSQI scoring was resolved by consensus and arbitrated by a third reviewer if necessary. If a study reported more than one outcome, the one with the highest score was taken into account.

## Results

### Study design and population characteristics

**Figure 1.**
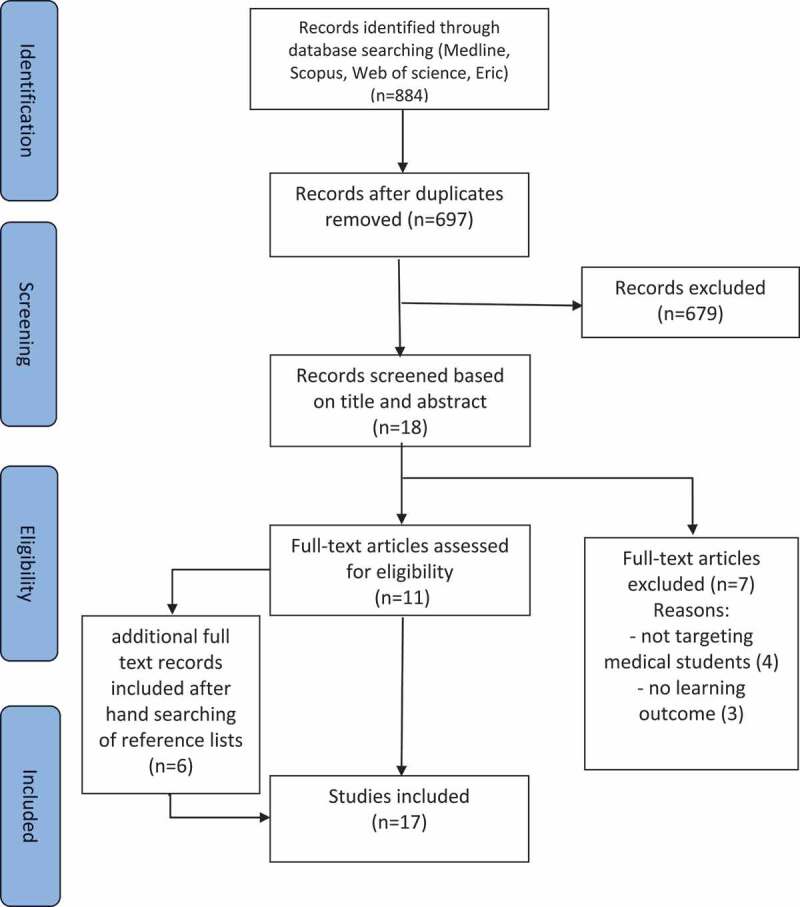
Flow-chart of the study selection.

Eight hundred eighty-four papers were identified after the initial databases search, of which 18 papers were retained after title and abstract screening (see Figure 1). Seven of them didn’t fit in the inclusion criteria for reasons as the absence of learning outcome or the targeted population being other than medical students. Finally, only 11 articles were retained, added to another 6 articles retrieved by cross-referencing. For the 17 articles included, the two reviewers agreed about 16 articles, and only one paper was discussed and decided to be included.

The 17 included papers reported 18 studies, as one paper included two distinct studies. Thirteen out of the eighteen studies were single group studies representing the most used study design (See Table 2). Eleven of these single group studies were cross-sectional while two were pre-post-test studies. The second most frequent study design encountered was cohorts, which were adopted in three studies. The remaining two were randomized controlled trials (RCT). The studies have been conducted between 1996 and 2019 with 13 studies (79%) from 2012 to 2019.

**Table 2.** Demographics, interventions, and outcome of the included studies

Author, year	Country	Study design	Participants	Subject	Intervention	Co-intervention	MCQtype	MCQ Quality	Instrument	Findings
Palmer Eet al. 2006 [10]	Australia	RCT	4th year N = 51	Surgery	Students were split randomly into 2 groups, the first group had to write a case report while the second had to write 3 MCQs in addition to the case report. Students were guided to write good MCQs	NR	NR	61% of MCQs: acceptable quality.Only 25% tested higher-order skills	Pre/post-test.	no significant difference
									Survey (learning methods ranking)	Both groups ranked learning methods similarly
	Australia	Single group cross-sectional	5th year N = 53	Diverse	Students were asked to rank their preferred learning methods before and after an activity in which they had to do research on a topic witha presentation and construct 3 MCQs for their peers to answer	NR	NR	Students created good quality MCQs.	Survey	a significant difference for the MCQ as a learning exercise option (p = 0.04) but the ranking among other activities remains poor
Chamberlain S et al. 2006 [43]	UK	Single-group cross-sectional	1st year N = 3, 2nd year N = 3	Diverse	After item-writing training Students worked singly or with peers to create (MCQs).students were getting paid to write MCQs	Feedback on each option given.	Type A	NR	Qualitative feedback	Students reported that the method helped them to consolidate their knowledge and comprehension of the curriculum
Gooi ACet al., 2014 [46]	Canada	Single-cross-sectional	1st year N = 113	Oto-laryngology	First step: Introductory session to how to write a high quality MCQs. Second step: Self-study sessionLast step: students created MCQs.MCQs were reviewed by instructors	Studentsreviewed each other’s MCQs	Type A	NR	Survey	Creating MCQs valuable = 78%.Reviewing MCQs valuable = 79% Class-created MCQs is a valuable resource = 91%Interested in collaborating in future session = 86%
Grainger Ret al. 2018 [30]	NewZealand	Singlegroup cross-sectional	4th year N = 106	Anatomic pathology	Students were instructed to write MCQs using bloom taxonomy and were required to write at least 2 in each module of anatomic pathology using PeerWise®	Rate and answer peer-generated MCQs, explain the answer and distractors.	Type A	74% of the MCQs were tested high order skills	Survey.	Students that were not satisfied with the process = 81%. Only 37% of students believed that interaction with peers was useful
Shah MPet al. 2019 [25]	USA	Single-group cross-sectional	2nd year N = 11	Cardio-pulmonary-renal	Students tooka workshop on how to write good quality MCQs and were asked to write MCQs on the lecture topics they were given	Giveexplanations of answers and distractors	NR	NR	Qualitative feedback	Studentsreported that creating MCQs and explaining answers helped them review key objectives and refined their test-taking strategies and would like to engage in similar activities
Walsh J et al. 2016 [44]	UK	Cohort	Final year (5th) N = 20.	Diverse	Students were instructed to write and create a bank of MCQs, Questions produced were arranged into a series of tests. the performance of question writers was compared to the rest of the students	Students had to meet for peer and senior clinician review of their MCQs	Type A	NR	Post-test	Students who wrote and reviewed questions scored higher than average compared to the rest of the students at the end of year summative exam (p ≤ 0.001)
Kurtz JB et al. 2019 [38]	USA	Single group cross-sectional	2nd yearN = 18	Cardiology	Participants were randomly divided into 6 groups of 3 students each, then students had to write 2 MCQs from exam blueprint subjects	Review MCQs with peers and faculty	Type A	NR	Qualitative analysis (n = 8).	Students found this activity beneficial on how to strategically approach MCQ examinations.Students voiced frustration about the time consumption
									Open-ended survey (n = 10).	Students found the activity beneficial for their learning (mean = 3.9 ± 0.3), They did not agree or deny that this was an efficient method to review cardiology (mean = 2.9 ± 1.1)
Benjamin HL et al. 2015 [47]	UK	Single group cross-sectional	5th year N = 20	Diverse	Students were asked to volunteer to create an online MCQ database, students had to write MCQs in a standard format, then the senior clinician was asked to review and approve MCQs	Students checked and reviewed each other’s MCQs	Type A	High quality	Mixed method feedback	100% positive feedback students reported question writing and/or peer review to be valuable for learning and useful for preparation and described it as enjoyable
Herrero JIet al. 2019 [26]	Spain	RCT	2nd & 3rd year N = 75 & N = 109	General pathology & Physiopathology	Students were invited to write 4 MCQs on a topic that was randomly chosen. They were offered an extra 0.25 point if their questions were good enough. The best 2 questions on each topic were selected to be included in the exam	NR	NR	Poor quality	Post-test	Students performed significantly better when writing MCQs on certain modules compared to others
Rajendiren S et al. 2014 [27]	India	Single-group Pre and post-test	First-year N = 135	Biochemistry	Students were classified into three categories: high, medium, and low performers. They took a pre-test, then the 3 different groups were given MCQ stems of the same subject and were asked to create distractors and the right answer separately, then they were tested again	NR	Distractors and answer	Good quality	Pre and post-tests.	A significant difference between pre and post-tests in both high and low performers
									Students feedback	55% of students found the exercise to be challenging and must be used as a learning exercise
Bobby Zet al. 2012 [48]	India	Single-group Pre and post-test	First-year N = 84	Biochemistry	Students took a pre-test then they were given 4 distractors in which one could be the answer. They were then asked to individually write the stem based on given keywords	Students engaged in small group discussions to review and modify MCQs of the group	Stems of MCQs	High quality	Pre and post-tests.	A significant difference between pre and the two post-tests in all students’ categories
									Students Feedback	95% of students wanted a second session in the future, 99% felt the exercise was not a burden
Sircar SSet al. 1999 [52]	India	Single-group cross-sectional	First-year N = 37	Physiology	A contest in which students had to write MCQs was organized. the contributors of the best and highest number of MCQs would be awarded certificates. And the best MCQs would be included in exams	NR	assertion-reasons MCQs	NR	Students Feedback	Most students agreed the contest was useful to their learning, though some found it time-consuming
McLeod PJ & Snell L. 1996 [28]	Canada	Single-group cross-sectional	2nd & 3rd year N = 150	Diverse	Students were divided into 3 groups, each one spent in rotation a 10 weeks clinical course. Each one was expected to write two to five MCQs. All accepted student-generated questions were included in the summative exams	NR	24% of MCQs were clinical case-based	Good quality	Students Feedback	Students appreciated how involved they were in the learning process. They recognized the benefits of reading when formulating a question
Papinczak T et al. 2012 [49]	Australia	Cohort	1st & 2nd year N = 384	Diverse	In small groups, students were asked to devote at least 1 hour a week to write MCQs type assertion-reason.MCQs and answers were reviewed by one or 2 academics before being loaded to a questions bank	Students were also asked to write short-answer & complex patient-based questions	assertion-reasonMCQs	Good quality	Pre and post-test.	Slight drop in grades
									Questionnaire	26% of students found the activity time consuming and challenging than expected. 77.3% supported the continuation of the project.
Stone MRet al. 2017 [31]	USA	Single-group cross-sectional	1st & 2nd year (N = 39).	Diverse	Students were asked to participate in crowd-sourced practice quizzes made of MCQs, matching, and true/false questions, based on the material taught. Each participant had to write questions for a certain number of lectures	Students took quizzes made of questions they generated	NR	Good quality	Post-test	No statistically significant difference between participants and non-participants. low performers benefited more from the process. Writing and taking tests were more effective than each one alone
									Survey	81.3% of students stated they felt more positive when they wrote MCQ
Walsh JLet al. 2018 [29]	UK	Cohort	1st & 2nd year N = 603	Diverse	PeerWise® was introduced to the first-year class of 2014. Over 2 years, students were asked to write MCQs.	Students were also asked to comment and rate their peers’ MCQs	NR	Acceptable quality	Post-test	There were significant correlations between writing, answering, and commenting frequency with summative examination performance (p < 0.001, R = 0.24, 0.13, and 0.15, respectively). PeerWise® users performed significantly better than non-users (p < 0.001)
									Students feedback	Students appreciated curricular specificity, and they were worried about the quality of student-authored questions
Jobs A et al. 2013 [45]	Germany	Single-group cross-sectional	4th year N = 102	Internal Medicine	Internal Medicine was divided into 4 sections which students had to take an exam on each. Students wrote MCQs 3 weeks before exams instructed by an approved manual, exam included some questions written by students	NR	Type A	Poor quality	Post-test	Low performers did significantly better while high performers didn’t have a measurable advantage
									Questionnaire	students spent less time designing MCQs compared to other methods. No apparent beneficial effects on learning habits

**MCQs**: Multiple-choice questions; **N**: Number; **NR**: Not reported; **RCT**: Randomized controlled trial

Regarding research methodology, 10 were quantitative studies, four were qualitative and four studies had mixed methods with a quantitative part and a qualitative one (students’ feedback).

Altogether, 2122 students participated in the 17 included papers. All participants were undergraduate medical students enrolled in the first five years of medical school. The preclinical stage was the most represented, with 13 out of the 17 papers including students enrolled in the first two years of medical studies.

Most studies used more than one data source, surveys were present as a main or a parallel instrument to collect data in eight studies. Other data sources were qualitative feedback (n = 8), qualitative feedback turned to quantitative data (n = 1), pre-post-test (n = 4), and post-test (n = 5).

### Quality assessment

Overall, the MERSQI scores used to evaluate the quality of the 14 quantitative studies were relatively above average which is 10.7, with a mean MERSQI score of 10.75, ranging from 7 to 14 (see details of MERSQI score for each study in Table 3). Studies lost points on MERSQI for using single group design, limiting participants to a single institution, the lack of validity evidence for instrument (only two studies used valid instrument) in addition to measuring the learning outcome only in terms of students’ satisfaction and perceptions.

**Table 3.** Methodological quality of included studies according to MERSQI

		Sampling			Validity evidence for evaluation instrument scores			Data analysis		Outcome (j)				
Authors	Study design (a)	Institutions studied (b)	Response rate (c)	Type of data (d)	Content (e)	Internal structure (f)	Relationships to other variables (g)	Appropriateness of analysis (h)	Complexity of analysis (i)	Satisfaction, Attitudes, perceptions	Knowledge,Skills	Behaviors	Patient/healthCare outcomes	Total
**Palmer E et al., 2006[**9**]**	3	0.5	1.5	3	1	0	0	1	2	-	1.5	-	-	13.5
	1	0.5	0.5	1	0	0	0	1	2	1	-	-	-	7
**Chamberlain S et al. 2006[**43**]**	-	-	-	-	-	-	-	-	-	-	-	-	-	-
**Gooi AC et al., 2014[**46**]**	1	0.5	1.5	1	0	0	0	1	1	1	-	-	-	7
**Grainger R et al. 2018[**30**]**	1	0.5	1	1	1	1	1	1	1	1	-	-	-	9.5
**Shah MP et al. 2019 [**25**]**	-	-	-	-	-	-	-	-	-	-	-	-	-	-
**Walsh J et al. 2016[**44**]**	2	0.5	1.5	3	1	0	0	1	2	-	1.5	-	-	12.5
**Kurtz JB et al. 2019[**38**]**	1	0.5	1.5	1	0	0	0	1	1	1	-	-	-	7
**Benjamin H. L. 2015 [**47**]**	1	0.5	1.5	1	0	0	0	1	1	1	-	-	-	7
**Herrero JI et al. 2019 [**26**]**	3	0.5	1.5	3	1	0	0	1	2	-	1.5	-	-	13.5
**Rajendiren S et al. 2014 [**27**]**	1.5	0.5	1.5	3	1	0	0	1	2	-	1.5	-	-	12
**Zachariah Bobby, 2012 [**48**]**	1.5	0.5	1.5	3	1	0	0	1	2	-	1.5	-	-	12
**Sircar SS et al. 1999[**52**]**	-	-	-	-	-	-	-	-	-	-	-	-	-	-
**McLeod PJ & Snell L. 1996 [**28**]**	-	-	-	-	-	-	-	-	-	-	-	-	-	-
**Papinczak T et al. 2012 [**49**]**	2	0.5	1.5	3	1	0	0	1	2	-	1.5	-	-	12.5
**Stone MR et al. 2017 [**31**]**	1.5	0.5	1.5	3	1	1	1	1	2	-	1.5	-	-	14
**Walsh JL et al. 2018[**29**]**	1.5	0.5	1.5	3	1	0	0	1	2	-	1.5	-	-	12
**Alexander Jobs, 2013 [**45**]**	1	0.5	1	3	1	0	0	1	2	-	1.5	-	-	11

***Details of MERSQI Scoring***:

a. Study design: Single group cross-sectional/post-test only (1); single group pre- and post-test (1.5); nonrandomized 2 groups (2); randomized controlled experiment (3).

b. Sampling: Institutions studied: Single institution (0.5); 2 institutions (1); More than 2 institutions (1.5).

c. Sampling: Response rate: Not applicable (0); Response rate < 50% or not reported (0.5); Response rate 50–74% (1); Response rate > 75% (1.5).

d. Type of data: evaluation by study participants (1); Objective measurement (3).

e. Validity evidence for evaluation instrument scores: Content: Not reported/ Not applicable (0); Reported (1).

f. Validity evidence for evaluation instrument scores: Internal structure: Not reported/ Not applicable (0); Reported (1).

g. Validity evidence for evaluation instrument scores: Relationships to other variables: Not reported/ Not applicable (0); Reported (1).

h. Appropriateness of analysis: Inappropriate (0); appropriate (1)

i. Complexity of analysis: Descriptive analysis only (1); Beyond descriptive analysis (2).

j. Outcome: Satisfaction, attitudes, perceptions (1); Knowledge, skills (1.5); Behaviors (2); Patient/health care outcome (3)

### Findings

The evaluation of the educational effect of MCQs writing was carried out using objective measures in 9 out of the 18 studies included, based on pre-post-tests or post-tests only. Subjective assessments as surveys and qualitative feedbacks were present as second data sources in 7 of these 9 studies, whereas they were the main measures in the remaining nine studies. Hence, 16 studies assessed the learning outcome in terms of students’ satisfaction and perceptions towards the activity representing the first learning level of the Kirkpatrick model which is a four-level model for analyzing and evaluating the results of training and educational programs [24]. Out of these 16 studies, 3 studies wherein students expressed dissatisfaction with the process and found it disadvantageous compared to other learning methods, whereas 4 studies found mixed results as students admitted the process value though they doubted its efficiency. On the other hand, nine studies provided favorable results of the exercise which was considered of immense importance and helped students consolidate their understanding and knowledge, although students showed reservations about the time expense of the exercise in three studies.

Regarding the nine studies that used objective measures to assess students’ skills and knowledge, which represent the second level of the Kirkpatrick model, six studies reported a significant improvement in students’ grades doing this activity, whereas two studies showed no noticeable difference in grades, and one showed a slight drop in grades.

One study suggested that students performed better when writing MCQs on certain modules compared to others. Two studies found the activity beneficial to all students’ categories while another two suggested the process was more beneficial for low performers.

Four Studies also found that writing and peer review combinations were more beneficial than solely writing MCQs. On the other hand, two studies revealed that peer-reviewing groups didn’t promote learning and one study found mixed results.

Concerning the quality of the generated multiple-choice questions, most studies reported that the MCQs were of good or even high quality when compared to faculty-written MCQs, except for two studies where students created MCQs of poor quality. However, only a few studies (n = 2) reported whether students wrote MCQs that tested higher-order skills such as application and analysis or simply tested recalling facts and concepts.

The majority of interventions required students to write single best answer MCQs (n = 6), three of which were vignettes MCQs. Assertion reason MCQs were present in two studies, and in one study, students were required to write only the stem of the MCQ, while in another study, students were asked to write distractors and the answer, while the rest of studies did not report the MCQs Type.

## Discussion

### Data and methodology

This paper methodically reviewed 17 articles investigating the impact of writing multiple-choice questions by medical students on their learning. Several studies pointedly examined the effect of the activity inquired on the learning process, whereas it only represented a small section of the article, which was used for the review. This is due to the fact that many papers focused on other concepts like assessing the quality of students generated MCQs or the efficiency of online question platforms, reflecting the scarce research on the impact of a promising learning strategy (creating MCQs) in medical education.

The mean MERSQI score of quantitative studies was 10.75 which is slightly above the level suggestive of a solid methodology set to 10.7 or higher [21]. This indicates an acceptable methodology used by most of the studies included. Yet, only two studies [30,31] used a valid instrument in terms of internal structure, content, and relation to other variables, making the lack of the instrument validity, in addition to the use of a single institution and single group design, as the main identified methodological issues.

Furthermore, the studies assessing the outcome in terms of knowledge and skills scored higher than the ones appraising the learning outcome regarding perception and satisfaction. Hence, we recommend that future research should provide more details on the validity parameters of the assessment instruments, and also focus on higher learning outcome levels; precisely skills and knowledge as they are typically more linked with the nature of the studied activity.

### Relation with existing literature

Apart from medical education, the impact of students’ generated questions has been a relevant research question in a variety of educational environments. Fu-Yun & Chun-Ping demonstrated through hundreds of papers that student-generated questions promoted learning and led to personal growth [32]. For example, in Ecology, students who were asked to construct multiple-choice questions significantly improved their grades [33]. Also, in an undergraduate taxation module, students who were asked to create multiple-choice questions significantly improved their academic achievement [34].

A previous review explored the impact of student-generated questions on learning and concluded that the process of constructing questions raised students’ abilities of recall and promoted understanding of essential subjects as well as problem-solving skills [35]. Yet, this review gave a general scope on the activity of generating questions, taking into consideration all questions formats. Thus, its conclusions will not necessarily concord with our review because medical students define a special students’ profile [36], along with the particularity of multiple-choice questions. As far as we know, this is the first systematic review made to appraise the pedagogical interest of the described process of creating MCQs in medical education.

### Students’ satisfaction and perceptions

Students’ viewpoints and attitudes toward the MCQ generation process were evaluated in multiple studies, and the results were generally encouraging, despite a few exceptions where students expressed negative impressions of the process and favored other learning methods over it [4,10]. The most pronouncing remarks were essentially on the time-consumption limiting the process efficiency. This was mainly related to the complexity of the task given to students who were required to write MCQs in addition to other demanding assignments.

Since the most preferred learning method for students is learning by doing, they presumably benefit more when instructions are conveyed in shorter segments, and when introduced in an engaging format [37]. Thus, some researchers tried more flexible strategies as providing the MCQs distractors and asking students for the stem or better providing the stem and requesting distractors as these were considered to be the most challenging parts of the process [38].

Some authors used online platforms to create and share questions making the MCQs generation smoother. Another approach to motivate students was including some generated MCQs in examinations, to boost students’ confidence and enhance their reflective learning [39]. These measures, supposed to facilitate the task, were perceived positively by students.

### Students’ performance

Regarding students’ performance, MCQs-generation exercise broadly improved students’ grades. However, not all studies have reported positive results. Some noted no significant effect of writing MCQs on students’ exam scores [10,31]. This was explained by the small number of participants, and the lack of instructors’ supervision. Moreover, students were tested on a broader material than the one they were instructed to write MCQs on, meaning that students might have effectively benefited from the process if they created a larger number of MCQs covering a wider range of material or if the process was aligned with the whole curriculum content. Besides, some studies reported that low performers benefited more from the process of writing MCQs, concordantly with the findings of other studies which indicate that activities promoting active learning advantage lower-performing students more than higher-performing ones [40,41]. Another suggested explanation was the fact that low achievers tried to memorize student-generated MCQs when these made part of their examinations, reversely favoring surface learning instead of the deep learning anticipated from this activity. This created a dilemma between enticing students to participate in this activity and the disadvantage of memorizing MCQs. Therefore, including modified student-generated MCQs after instructors’ input, rather than the original student-generated version in the examinations’ material, might be a reasonable option along with awarding extra points when students are more involved in the process of writing MCQs.

### Determinant factors

Students’ performance tends to be related to their ability to generate high-quality questions. As suggested in preceding reviews [35,42], assisting students in constructing questions may enhance the quality of these students’ generated questions, encourage learning, and improve students’ achievement. Also, guiding students to write MCQs makes it possible to test higher-order skills as application and analysis besides recall and comprehension. Accordingly, in several studies, students were provided with instructions on how to write high-quality multiple-choice questions, resulting in high-quality student-generated MCQs [10,43–45]. Even so, such guidelines must take into account not making students’ job more challenging to maintain the process as pleasant.

Several papers discussed various factors that influence the learning outcome of the activity, as working in groups and peer checking MCQs, which were found to be associated with higher performance [30,38,43,44,46–49]. These factors were also viewed favorably by students because of their potential to broaden and deepen one’s knowledge, as well as to notice any misunderstandings or problems, according to many studies, that highlighted a variety of beneficial outcomes of peer learning approaches in the education community [42,50,51]. However, in other studies, students preferred to work alone and demanded that time devoted to peer-reviewing MCQs be reduced [38,45]. This was mostly due to students’ lack of trust in the quality of MCQs created by peers; thus, evaluating students’ MCQs by instructors was also a component of an effective intervention.

### Strengths and limitations

The main limitation of the present review is the scarcity of studies in the literature. We used a narrowed inclusion criterion leading to the omission of articles published in non-indexed journals and papers from other health-care fields that may have been instructive. However, the choice of limiting the review scope to medical students only was motivated by the specificity of the medical education curricula and teaching methods compared to other health professions categories in most settings. Another limitation is the weak methodology of a non-negligible portion of studies included in this review which makes drawing and generalizing conclusions a delicate exercise. On the other hand, this is the first review to summarize data on the learning benefits of creating MCQs in medical education and to shed light on this interesting learning tool.

## Conclusion

Writing multiple-choice questions as a learning method might be a valuable process to enhance medical students learning despite doubts raised on its real efficiency and pitfalls in terms of time and effort.

There is presently a dearth of research that examines the influence of student-generated MCQs on learning. Future research on the subject must use a strong study design, valid instruments, simple and flexible interventions, as well as measure learning based on performance and behavior, and explore the effect of the process on different students’ categories (eg. performance, gender, level), in order to reach the most appropriate circumstances for the activity to get the best out of it.

## Appendix: Search strategy

- **Medline**:
  - Query: ((((Medical student) OR (Medical students)) AND (((Create) OR (Design)) OR (Generate))) AND ((((multiple-choice question) OR (Multiple-choice questions)) OR (MCQ)) OR (MCQs))) AND (Learning)
  - Results: **300**
- **Scopus**:
  - Query: ALL (medical PRE/0 students) AND ALL (multiple PRE/0 choice PRE/0 questions) AND ALL (learning) AND ALL (create OR generate OR design)
  - Results: **468**
- **Web of science**:
  - Query: (ALL = ‘Multiple Choice Questions’ OR ALL = ‘Multiple Choice Question’ OR ALL = MCQ OR ALL = MCQs) AND (ALL = ‘Medical Students’ OR ALL = ‘Medical Student’) AND (ALL = Learning OR ALL = Learn) AND (ALL = Create OR ALL = Generate OR ALL = Design)
  - Results: **109**
- **ERIC**:
  - Query: ‘Medical student’ AND ‘Multiple choice questions’ AND Learning AND (Create OR Generate OR Design)
  - Results: **7**

Total = 884

**After deleting double references**: Number: 697
